# Hospitals and Country Annexes

**Published:** 1923-12

**Authors:** 


### HOSPITALS AND COUNTRY ANNEXES.

The opening of the Catherine Gladstone Home, Mitcham, as an annexe of the London Hospital, which took place on November 19, was an occasion of real interest to all interested in the future of our hospitals and their maintenance as voluntary institutions. The Home was founded by the late Mrs. W. E. Gladstone for children who lost both their parents in the cholera epidemic of 1866, and was originally established at Woodford. The Institution gradually changed in character to a general Convalescent Home, and was removed to its present situation at Mitcham.

### An Annexe to the "London."

At the end of 1922, together with the endowment amounting to approximately £20,000, it was handed over to the London Hospital for use as a country annexe—that is, as a ward of a hospital outside the main building. The establishment of such outside wards of the hospital is an attempt to intensify the service of the hospital beds to the community. By this means the cost of maintenance will be reduced, as the diseases of patients will be diagnosed at the central hospital and operations performed there; the patients will then as soon as possible be removed to the annexe to complete their recovery, and their beds will be free for the reception of other patients.

### Enter "Marie Céleste."

When the generous offer of the Home and its endowment was made to the London Hospital, the House Committee were unable at first to accept, as they had not the wherewithal necessary for the maintenance of the Home. At this juncture, however, they received an offer of help from the "Marie Céleste" Samaritan Society. This Society, which is the oldest of its kind, was founded in 1791 by Mr. James Hora, whose wife bore the name of Marie Céleste. The Society offered to undertake the cost of altering and adapting the Home, to bear the cost of furniture and equipment, and also to provide means for keeping up the Home over and above the amount which was supplied by the endowment.

### Curing in the Country.

Sir William Joynson-Hicks, Minister of Health, at the opening ceremony emphasised the great advance in hospital management represented by this scheme, and said he trusted that it might be the pioneer of many annexes, especially in London and other large cities. He hoped the day would come when the central hospitals might devote their energies to scientific and diagnosis work, and the operations and cures take place in the country annexes. Lord Knutsford pointed out that the whole future treatment of hospital patients was concerned, and as experiments with annexes in connection with the London Hospital had already been tried with marked success, he anticipated that the Catherine Gladstone Annexe would still further demonstrate the success of this particular form of hospital treatment. The Home as rearranged gives accommodation for 42 patients, and no expense has been spared to equip it in a practical and modern way. It may well serve as a model for other hospitals desirous of starting an annexe on similar lines.

